# A Blur Feature-Guided Cascaded Calibration Method for Plenoptic Cameras

**DOI:** 10.3390/s25164940

**Published:** 2025-08-10

**Authors:** Zhendong Liu, Hongliang Guan, Qingyang Ni

**Affiliations:** 1 College of Resource Environment and Tourism, Capital Normal University, Beijing 100048, China; lzdgis@casm.ac.cn (Z.L.); 1240921021@cnu.edu.cn (Q.N.); 2 Engineering Research Center of Spatial Information Technology, Beijing 100048, China; 3 Chinese Academy of Surveying and Mapping, Beijing 100830, China

**Keywords:** light field camera calibration, multi-focus configuration, blur-aware feature, geometric observation of virtual depth space, stepwise diffusion model, decoupled stacking optimization

## Abstract

Accurate and robust calibration of multifocal plenoptic cameras is essential for high-precision 3D light field reconstruction. In this work, we propose a blur feature-guided cascaded calibration for the plenoptic camera. First, white images at different aperture values are used to estimate the high-confidence center point and radius of micro-images, and the defocus theory is used to estimate the initial values of the intrinsic parameters. Second, the gradient value is introduced to quantify the degree of blurring of the corner points, which are then divided into three types: clear, semi-clear, and blurred. Furthermore, a joint geometric constraint model of epipolar lines and virtual depth is constructed, and the coordinates of the semi-clear and blurred corner points are optimized in a step-by-step manner by using the clear corner point coordinates. The micro-image center ray projection equation is then devised to assist in the optimization of the microlens array core parameters and establish blur-adaptive credibility weights, thereby constructing a global nonlinear optimization. Finally, the proposed method is tested on both simulated and captured datasets, and the results exhibit superior performance when compared with the established methods described by Labussière, Nousias, and Liu. The proposed method excels in corner feature extraction, calibration accuracy of both internal and external parameters, and calibration sensitivity when applied to multifocal-length light field cameras, highlighting its advantages and robustness.

## 1. Introduction

Plenoptic (light field) imaging can record the position and direction of light in a scene, forming a light field image containing four-dimensional information. By using this information, sub-aperture images at different angles can be obtained, or refocusing can be used to obtain refocused images with different depths of field [1,2,3]. These characteristics give light field imaging technology unique advantages in terms of depth estimation and 3D reconstruction, and it is gradually being incorporated into autonomous driving, digital twins, augmented reality, industrial inspection, and medical imaging techniques [3,4,5].

Camera calibration is a process that determines the intrinsic and extrinsic parameters of a camera and establishes an accurate correspondence between image pixels and real-world coordinates. Defining this correspondence is a fundamental problem in computer vision, an important basis for reconstruction and positioning, and an important bridge for establishing connections between 2D images and 3D space [5]. On the basis of camera design structures, the existing calibration methods can be roughly divided into those involving camera arrays [4,5,6,7], unfocused light field cameras [8,9,10,11], and focused light field cameras [12,13,14,15]. Among them, the core research on light field camera calibration has focused on imaging models and the accuracy and robustness of the calibration parameters. However, for more advanced multifocal focused light field cameras designed for varying working distances (such as small scenes at close range and large scenes at long range), the existing methods present very significant challenges in terms of the accuracy and sensitivity of the calibration parameters.

To address these problems, we propose a blur feature-guided cascaded calibration method for plenoptic cameras (BFC-PC). The main contributions of this paper are as follows:

1. For the first time, a geometric observation model of the virtual depth domain is proposed. The geometric relationship between the epipolar lines in the micro-image (MI) and the virtual depth space is used to construct a geometric observation model of the virtual depth domain that is unique to plenoptic cameras.
2. A quantification and grading rule for micro-image clarity that accounts for blur-perceived plenoptic features is constructed. The geometric observation mode of the virtual depth domain is used to iteratively filter the outliers and optimize the corner point coordinates in a step-by-step diffusion manner, effectively improving the extraction quality of the corner point features.
3. A decoupled and cascaded optimization strategy is proposed for the first time. The central light projection equation of the target MI is constructed to assist in optimizing the parameters of the microlens array (MLA), and redundant observations of the same corner point and adaptive confidence weights that account for blur are established to construct a global nonlinear optimization model. The complexity of the nonlinear function can thus be effectively reduced, avoiding sensitivity to the initial estimated parameters and making it easier to converge to the optimal solution.

The components of this work are as follows: Section 2 introduces the existing focused light field camera calibration methods and their shortcomings, Section 3 details the methods proposed in this paper, Section 4 presents the experiments and analyses of the results, and Section 5 presents the conclusions.

## 2. Related Work

The related work in this field has centered primarily on focused light field camera-based approaches. According to the different types of images used during the calibration process, the existing focused light field camera calibration methods can be roughly divided into two categories: calibration methods based on interpreted images and calibration methods based on original images.

Calibration methods based on interpreted images rely on reconstructed images (such as sub-aperture images and all-focus images). Johansend et al. [12] first proposed a focused light field camera calibration method and introduced a new model for correcting radial and depth distortions; however, this approach easily falls into local optima and requires a good initial value to converge. Heinze et al. [16] further improved the model and incorporated the main lens tilt and displacement into the model, and the results were incorporated into the RxLive software from Raytrix GmbH. Zeller et al. [13,17] proposed two methods for calibrating focused plenoptic cameras and depth maps. They extracted features from the input fully focused image and equivalently treated the focused light field camera as a multi-eye pinhole camera array for camera calibration purposes.

Some scholars have proposed the direct use of original light field images to calibrate camera parameters. Noury et al. [15] defined the projection process from a spatial point to a raw light field image; at the same time, a new corner detection algorithm was developed to obtain the corners from the raw image with subpixel accuracy. O’Brien et al. [18] proposed the concept of circular-domain light field features, which were used to determine the correspondences between corners, and Liu et al. [19] used a step-by-step method to detect the corner features of a raw image and complete the calibration process. Nousias et al. [14] established a projection model from spatial points to original images and developed a new corner detection algorithm to extract checkerboard corners. Furthermore, Labussière et al. [20,21] calibrated the focal length of the MLA; however, the accuracy of the blur radius was low. The calibration method used for a focused light field camera usually includes three key steps: initial estimation of the calibration parameters, feature extraction, and nonlinear optimization.

In recent years, deep learning-based approaches for corner detection and adaptive weighting have shown promising advances in camera calibration tasks. For example, CCDN (Checkerboard Corner Detection Network) [22] introduces a fully convolutional network tailored for detecting corner points under distortion, noise, and blur, achieving high robustness across challenging imaging conditions. Although such methods perform well on conventional cameras, they are not specifically designed for the multi-aperture and defocus characteristics of light field imaging. Additionally, several learning-based camera calibration frameworks have been proposed. LiFCal [23] presents an online calibration strategy for light field cameras by leveraging bundle adjustment and dynamic sequence analysis, enabling real-time parameter estimation. Moreover, Liao et al. [24] provide a comprehensive survey of deep learning approaches for camera calibration, including techniques involving uncertainty modeling and adaptive confidence weighting.

While these learning-based strategies offer automation and robustness, most of them focus on standard image modalities and lack physical interpretability in plenoptic camera geometry. In contrast, our method proposes a blur-aware corner modeling and cascaded optimization strategy that explicitly leverages the multi-focus nature of plenoptic images, offering better integration with the physical imaging model and higher controllability for calibration tasks.

When calibrating a multifocal focused light field camera, the feature extraction and nonlinear optimization steps still present problems such as feature points with uneven quality levels, highly complex optimization problems, and high sensitivity to the initial estimation value.

(1) Feature point quality issues

Currently, the corner detectors used in more advanced methods include saddle point detection methods [14] and template matching methods [15,20]. The primary conception behind saddle point detection methods is that when a corner point is present in an MI, that corner point is found at the “saddle point/maximum and minimum axes” in the intensity domain. These two axes are not necessarily orthogonal, but the direction of the saddle is determined by using the corner point as the center, where these are the maximum and minimum cumulative intensity lines passing through the image. These lines determine two potential saddle axes, and the corner point is located at their intersection. Moreover, the main idea motivating template matching methods involves describing the appearance characteristics of a corner point (such as its intensity distribution or geometric shape) via a predefined template, adjusting the template parameters and calculating similarity values (such as the cross-correlation and mean squared error metrics) to best fit the target corner point and calculate its coordinate value.

For a certain corner point in the real 3D world, the MIs formed by a multifocal focused light field camera will exhibit different degrees of blur. The existing corner detectors all use the characteristics of the image intensity changes to extract corner points, and when an MI is relatively blurred, the intensity change induced at the corner point is distributed in a pixel area within a certain range centered on the real corner point. Therefore, the corner point coordinates extracted by the existing corner detectors are inaccurate or even wrong, which leads to poor accuracy for the calculated camera parameters. Notably, the greater the blur of the MI is, the larger the intensity change distribution area, and the worse the accuracy of the extracted corner point coordinates may be.

(2) Sensitivity of nonlinear optimization to initial values

Nonlinear optimization involves optimizing the internal and external camera parameters of the initial estimation process to minimize the reprojection error. The existing methods typically use nonlinear optimization algorithms such as the Levenberg–Marquardt algorithm [25] or the Powell method [26] to optimize all of the parameters at once. These nonlinear optimization algorithms usually rely on the initial value to gradually adjust their parameters and iteratively calculate them to approach the optimal solution of the objective function. However, this single process for optimizing all of the parameters may be sensitive to the selected initial value. The main reasons for these findings include the following three points: First, due to the complexity of nonlinear functions, different initial values may cause the employed nonlinear optimization algorithm to iterate along different paths and finally converge to different solutions. Second, multiple extreme points may be present in a nonlinear optimization problem, including local optimal and global optimal values. If the initial point is not selected properly, the algorithm may fall into a local optimal solution and fail to find the global optimal solution because the nonlinear function may have a flat gradient or steep curvature near a local optimal solution, making it difficult for the algorithm to jump out of the local optimal area. Finally, if the initial point is not chosen properly, a nonlinear optimization algorithm may not converge to the optimal solution during the iteration process because of the accumulation of factors such as calculation errors and rounding errors.

## 3. Methods

**Figure 1:**
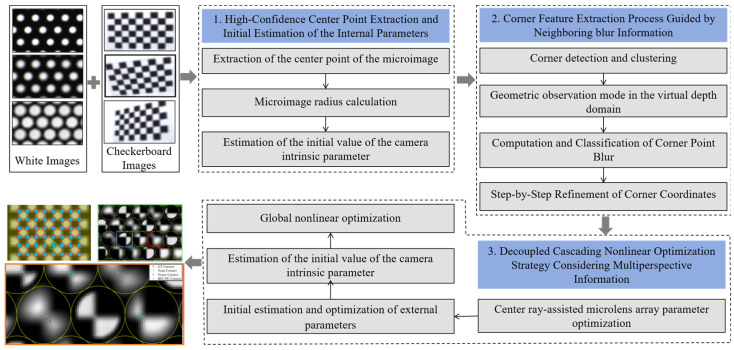
Overview of the methodology proposed in this paper.

When calibrating multifocal plenoptic cameras, we overcome accuracy and sensitivity issues via three streamlined stages: (1) Robust Initialization of the Intrinsic Parameters: Multiple white images corresponding to different aperture values are used to estimate the center and radius of the target MI. The linear relationship between the aperture and radius is fitted according to defocusing theory to solve for the initial values of the intrinsic parameters. (2) Blur-Guided Corner Extraction: In this process, the raw checkerboard corners are detected and clustered and then sorted by the estimated blur. The outliers are then filtered via epipolar and virtual depth constraints, and the coordinates are iteratively refined from sharp to blurred by employing a diffusion strategy. (3) Cascaded, Decoupled Optimization: First, the microlens array geometry is refined with a central-ray projection model. Then the intrinsic parameters are fixed, and the extrinsic parameters are estimated via a pinhole-lens model by using the clearest corners. Finally, gross corner errors across frames are eliminated, and global blur-weighted nonlinear optimization is performed. An overview of the proposed method is shown in Figure 1.

### 3.1. Micro-Image Central Grid and Initial Intrinsic Parameter Estimation

The high precision of micro-image central grid parameters and initial estimated intrinsic parameter values are related to the accuracy of the geometric observation data, namely, the original white image (generally obtained via light diffuser photography), and existing methods usually use only a single white image at different apertures to extract the center point and micro-image radius. However, since it is impossible to strictly control photographic conditions when shooting white images, there may be overexposure or underexposure in addition to large errors due to improper operation when the camera aperture scale value is manually adjusted. Ultimately, the center point and micro-image radius extracted from the original white image may have large errors. The proposed method uses white images taken at different apertures to extract the coordinates of the center point and estimate the initial values of the intrinsic parameters. Its accuracy directly affects the geometric constraint reliability of the virtual depth observation mode and the convergence efficiency of the decoupled stacking optimization (reasonable initial parameter values are required).

**Figure 2:**
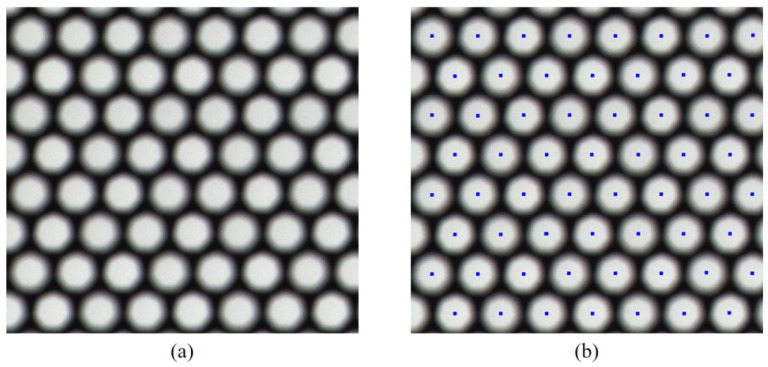
Results of center extraction. (**a**) White image and (**b**) center of the micro-image.

**Extraction of the center point of the micro-image**. Due to the vignetting effect at the edge of the microlens, the brightest position of the micro-image in the white image can be regarded as the center of the microlens above it. The white image is processed via brightness correlation and non-maximum suppression, and the position of the maximum light intensity in each micro-image is determined by finding the micro-image center with the subpixel precision intensity centroid method [27,28]. Figure 2 shows the estimated distribution of the micro-image center.

First, by using the different focal lengths of the microlenses and changing the aperture of the main lens, the camera aperture is adjusted to different scale values $N_{m},\;m\in \{0,1,\ldots ,M\}$ via a light diffuser mounted on the main lens, and multiple original white images $I_{j},\;j\;\in \{0,\;1,\ldots ,J\}$ are taken at the same scale value. Second, the subpixel precision intensity centroid method [15,27,28] is used to detect the micro-image center $C_{k,l},(k,l)\in \{(0,\;0),(0,\;1),\;\ldots ,(K,L)\}$ in all original white images at a certain aperture scale value $N_{m}$. To avoid outliers at the center point of the extracted micro-image caused by the randomness of the above-mentioned errors, the median of the center of the micro-image under the aperture scale value $N_{m}$ is obtained by calculating the median of the relationship between the multiple original white images and the micro-image center $\tilde{C_{k,l}},(k,l)\in \{(0,\;0),(0,\;1),\;\ldots ,(K,L)\}$. The pixel translation $\left(T_{x},T_{y}\right)$ and rotation angle φ of the micro-image array are calculated via a nonlinear optimization method that uses the extracted median coordinates of all of the micro-images.

After the coordinates of the micro-image center are obtained, the micro-image center grid parameters are then fitted. First, the initial side length of the micro-image array is determined by calculating the distance between adjacent micro-image centers, and then, according to the MLA arrangement rule, the coordinate position $C_{k,l}^{*}={\left[u,\;v,\;1\right]}^{T}$ of the grid vertex on the image plane can be expressed as

(1) $$Row-aligned:\left\{\begin{array}{c} \tau_{offset}=\frac{1}{2}\;(if\;l\;is\;even\;,\;0\;otherwise)\\ u=\delta_{i}\cdot (k+\tau_{offset})\\ v=\delta_{i}\cdot l\cdot sin\frac{\pi }{3} \end{array}\right.\;Column-aligned:\left\{\begin{array}{c} \tau_{offset}=-\frac{1}{2}\;(if\;k\;is\;odd,\;0\;otherwise)\\ u=\delta_{i}\cdot k\cdot cos\frac{\pi }{6}\\ v=\delta_{i}\cdot (l+\tau_{offset}) \end{array}\;\right.$$

where (k, l) represents the row and column number of the vertex in the grid and where $\tau_{offset}$ is the offset.

Finally, the Levenberg–Marquardt algorithm is used for nonlinear optimization to compare the distances between the mesh vertices and the detected micro-image center to determine the pixel offset $\left(T_{x},\;T_{y}\right)$ in the image coordinates and the rotation angle $\vartheta_{z}$ around the *Z*-axis:

(2) $$\arg\underset{\left\{\tau_{x},\tau_{y},\vartheta_{z},\delta_{i}\right\}}{min}\;\sum_{\left(k,l\right)}\;{∥Tc_{k,l}^{*}-c_{k,l}∥}^{2}with\;T=\left[\begin{array}{ccc} cos\vartheta_{z} & -sin\vartheta_{z} & \tau_{x}\\ sin\vartheta_{z} & cos\vartheta_{z} & \tau_{y}\\ 0 & 0 & 1 \end{array}\right]$$

**Figure 3:**
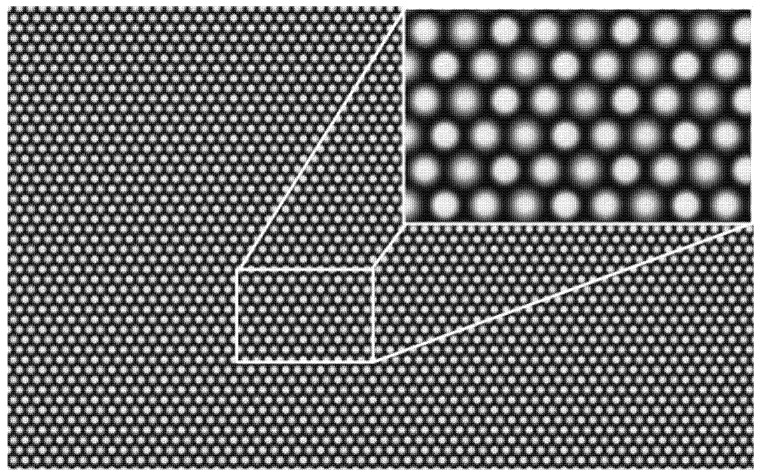
White image captured by a multifocal light field camera.

**Estimation of the micro-image radius**. In a white image, different types of MLAs produce micro-image features with specific sizes and light intensity distributions, as shown in Figure 3. For all original white images of size $N_{m}$ at the same aperture scale, the Euclidean distance D(k, l) between the micro-image center $C_{k,l}$ and the median $\tilde{C_{k,l}}$ corresponding to each MLA $\left(k,\;l\right)$ is calculated, and the MLA is degenerated into a grid image to generate an error map, where one pixel represents one MLA and the pixel value is D(k, l) in the normalized range of 0 to 255. The structural similarity index proposed is then used to evaluate the similarity between the error maps by comparing the pixel values, brightness, contrast, and structural information. The error maps with lower similarity are selected, and the corresponding original white images are regarded as unreliable data sources in terms of calculating the micro-image radius. To avoid the influence of the vignetting effect, the micro-image radius is estimated only for the micro-image in the center area of the white image. For the white image $I_{j}$ of a certain aperture scale value $N_{m}$, the second-order central moment is calculated by the median of the micro-image center, the covariance matrix is constructed, and the micro-image radius is calculated, according to reference [20].

**Estimation of the initial intrinsic parameters**. By using the correspondence $N=\sqrt{2^{AV}}$ between the lens aperture value AV and the actual aperture value N, the actual aperture value N corresponding to each white image can be calculated. Then, according to the linear relationship between the micro-image radius R and the actual aperture value N, the linear parameters are calculated as follows:

(3) $$R_{i}(N^{-1})=m\cdot \;N^{-1}+q_{i}$$

where i represents the type of MLA, $R_{i}$ represents the micro-image radius of type i, and *m* and $q_{i}$ are linear parameters. The intrinsic parameter relationships are defined in Formulas (4) and (5):

(4) $$m=\frac{d\cdot F}{2D}$$

(5) $$q_{i}=\frac{1}{f_{i}}(\frac{∆c\cdot D}{d+D})\cdot \frac{d}{2}-\frac{∆c}{2}$$

where d represents the distance from the MLA to the sensor, F represents the focal length of the main lens, D represents the distance from the MLA to the main lens, $f_{i}$ represents the focal length of different types of MLAs, and ∆c represents the diameter of the micro-image.

The initial intrinsic parameters of the camera are further calculated by using the *m* and $q_{i}$ values calculated above as well as the factory parameters of the camera (focal length F of the main lens and sensor size). The calculation formulas for parameters *d* and *D* are as follows:

(6) $$d=\frac{2m}{F+4m}\cdot \left|\frac{h}{2}\left(1-\sqrt{1-4\frac{F}{h}}\right)\right|$$

(7) $$D=\left|\frac{h}{2}\left(1-\sqrt{1-4\frac{F}{h}}\right)\right|-2d$$

**Figure 4:**
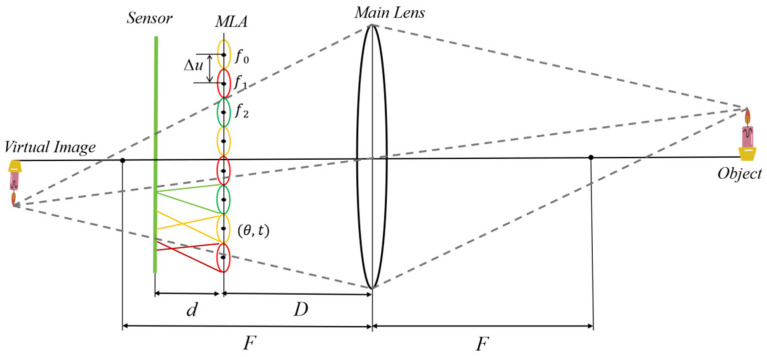
Initial values for estimating the intrinsic camera parameters. F represents the focal length of the main lens, D represents the distance from the MLA to the main lens, d represents the distance from the MLA to the sensor, ∆μ represents the spacing of the MLA, (θ,t) represents the rotation and translation of the MLA relative to the main lens, and $f_{0}$, $f_{1}$, $f_{2}$ represent three types of MLAs with different focal lengths.

We set the distance between the main lens plane and the sensor plane to (D+d) and the MLA plane to be parallel to the main lens plane, with a distance of D. Furthermore, we set the principal point to the center of the image and initialized all distortion coefficients of the main lens to zero. The translation $\left(t_{x},t_{y}\right)$ and rotation angle θ of the MLA are initialized by the pixel offset $\left(T_{x},T_{y}\right)$ and rotation angle φ of the acquired micro-image parameters, as shown in Figure 4. Among them, θ can be decomposed into three components, the rotation angles $\theta_{x}$, $\theta_{y}$, and $\theta_{z}$ around the *X*-axis, *Y*-axis, and *Z*-axis. The rotation angles around the *X*-axis and *Y*-axis are initialized to zero. The MLA focal length $f_{i}$ is initialized to

(8) $$f_{i}=\frac{d}{2\cdot q_{i}^{\prime }}\cdot \frac{D}{D+d}\cdot ∆c=\frac{d}{2\cdot q_{i}^{\prime }}\cdot ∆\mu \;$$

### 3.2. Corner Feature Extraction Based on Blur Quantization Under the Virtual Depth Geometric Constraint Model

The intensity change at the corner point in the micro-image in the original frame is distributed within a certain range of pixels centered on the real corner point. Notably, the greater the blur in the micro-image is, the larger the intensity change distribution area, and thus, the corner point coordinates extracted by the existing method will have errors or even mistakes.

To effectively improve the accuracy of the corner point coordinates, this paper proposes a corner point feature extraction strategy guided by neighboring blur information. This approach uses the geometric relationship between the epipolar lines between the micro-images and the virtual depth space to build a geometric observation model of the virtual depth domain; then, by using this constructed model, the degree of the blurred imaging at multiple corner points in the cluster can be determined, and the corner point coordinates can be optimized from clear to blurred in a step-by-step diffusion manner. This process proceeds as follows:

**Determination of the correspondence between corner points**. When a traditional optical camera performs imaging, a 3D point projected into a single-frame image will only form a 2D point. In contrast, since the focused light field camera adds an MLA between the main lens and the sensor, this optical path design decomposes the light of the 3D point into multiple light beams with different directions. These light beams form multiple 2D points in multiple micro-images, which are generally called points of the same name. Since the subsequent ambiguity calculations, joint geometric constraint model construction, and corner point optimization are applicable only to points of the same name, it is necessary to first determine the correspondence between these 2D corner points.

**Figure 5:**
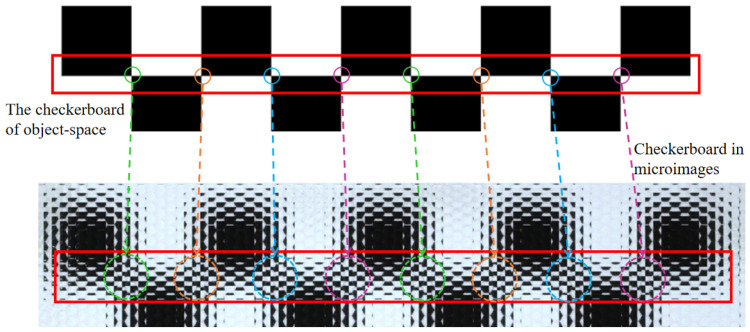
Correspondence between corner points in the object space and image space of a chessboard.

When a focused light field camera is used to capture the corners of a chessboard, the light emitted from the same corner in the object space is projected onto multiple adjacent image points on the sensor; the spacing between these image points is very close, whereas the corners of the image space projected onto the sensor from different corners in the object space are far apart, as shown in Figure 5.

**Figure 6:**
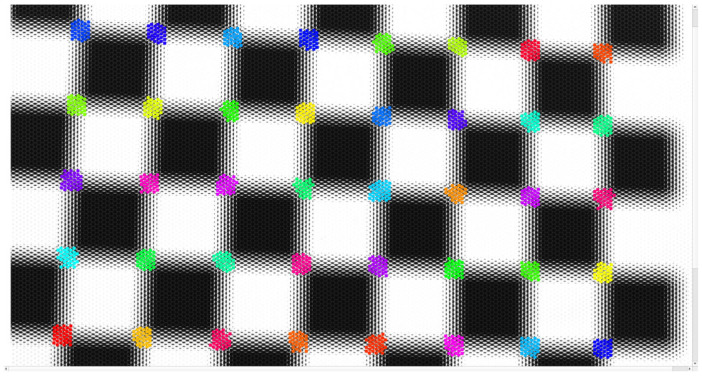
Corner clustering results, different colors represent different clusters.

Based on the distribution law for projected 2D corner points, to effectively identify and process these observations, the Density-Based Spatial Clustering of Applications with Noise (DBSCAN) [29] spatial clustering algorithm is used to cluster the corner point coordinates detected within a certain distance. All corner point coordinates in the cluster are regarded as the projection point coordinates for the same 3D point in the image. This clustering algorithm can not only effectively process the spatial distribution relationship between corner points but also reduce the mismatch caused by image blur or noise interference to a certain extent. The corner point clustering result is shown in Figure 6, where different colors represent different corner point clusters.

**Figure 7:**
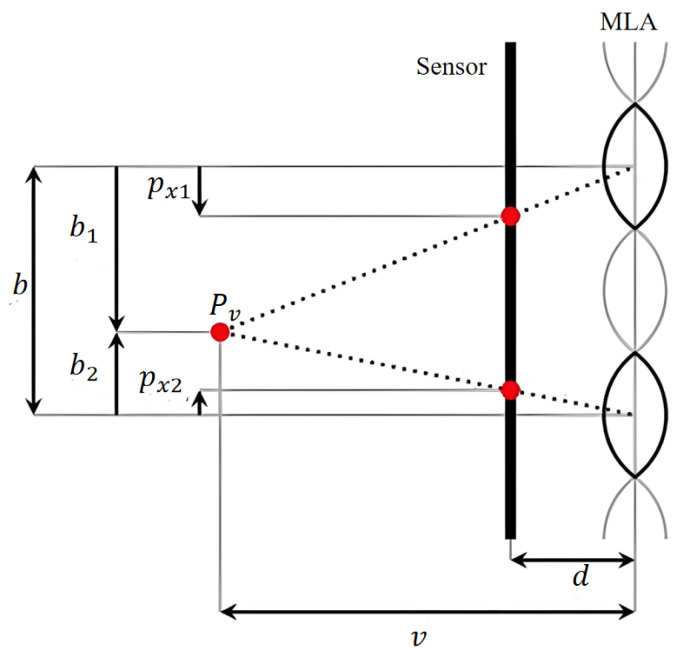
Geometric relationships between image points and virtual image points in the virtual depth domain.

**Geometric observation mode in the virtual depth domain**. Since MLAs of different focal lengths image the same corner point on a black and white chessboard, they will appear as corner points with different blur levels in the image space. Therefore, the coordinates of the corner points detected by existing methods are inaccurate. We analyze and utilize the intrinsic structure and imaging characteristics of multifocal focused light field cameras and propose a geometric observation mode in the virtual depth domain to improve the accuracy of the corner point coordinates and ensure the accuracy of camera parameter estimation, as shown in Figure 7.

In our model, both the Main Lens and the Microlens Array (MLA) are treated as thin lenses, consistent with classical geometric optics assumptions. As described by Zeller et al. [13], in both Galilean and Keplerian configurations of focused plenoptic cameras, the light rays refracted by the Main Lens tend to converge toward a specific spatial location. Although this location does not physically coincide with the sensor plane and cannot form a real image, it can be regarded as a virtual image point, which serves as a geometric reference for modeling. In this framework, a single object-space corner point is projected by the Main Lens onto a virtual image point, which is then observed by multiple microlenses from different viewpoints. These microlens observations produce multiple image-space corner projections that are constrained by a shared geometric relationship in the virtual depth domain.

Since all corner points in a cluster correspond to the same virtual image point, if the coordinates $I_{i}$ and $I_{j}$ of two or more corner points are known, the relative distance (virtual depth) v between the MLA and the virtual image can be calculated via triangulation.

(9) $$v=\frac{\left|M_{i}-M_{j}\right|}{\left|M_{i}-M_{j}\right|-\left|I_{i}-I_{j}\right|}$$

where $\left|M_{i}-M_{j}\right|$ represents the Euclidean distance (baseline) between the centers of MLA $M_{i}$ and $M_{j}$, $\left|I_{i}-I_{j}\right|$ represents the Euclidean distance between corner points $I_{i}$ and $I_{j}$ in the micro-image, $\left|M_{i}-M_{j}\right|-\left|I_{i}-I_{j}\right|$ is the parallax between corner points $I_{i}$ and $I_{j}$, and *i* and *j* represent the index numbers of the MLA and its micro-image, respectively.

Once the virtual depth *v* is estimated, a certain corner point $I_{i}$ can be used to backproject the virtual image point $P_{v}$ into the micro-image, where any corner point in the cluster is located by the following formula to obtain the back-projected coordinate $I_{j}^{\prime }$:

(10) $$I_{j}^{\prime }=I_{i}+\underset{C_{j}-C_{i}}{\to }\cdot (\left|M_{j}-M_{i}\right|-\frac{\left|M_{j}-M_{i}\right|}{v})\;\;$$

where $\underset{C_{j}-C_{i}}{\to }$ represents the unit direction vector from the micro-image center $C_{j}$ to the micro-image center $C_{i}$, that is, the epipolar direction.

**Figure 8:**
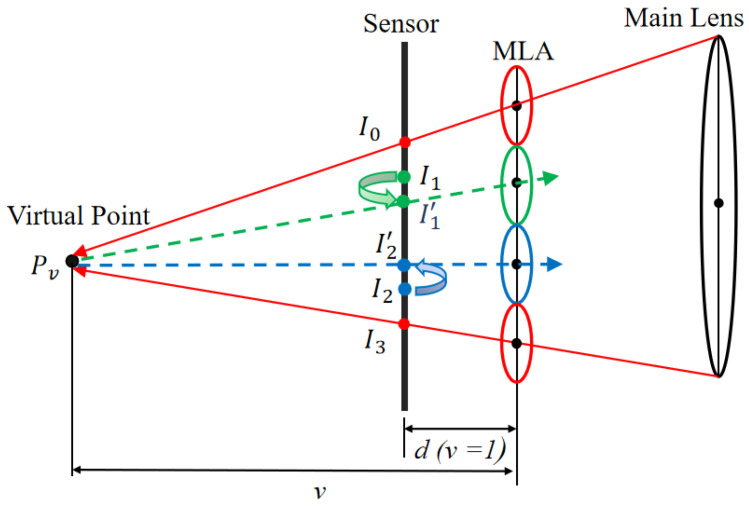
Geometric observation in the virtual depth domain. The geometric observation model for corners in clusters in the virtual depth domain is depicted, where I′1 is the corner of I1, corrected by the geometric observations of clear corners such as I0 and I3 in the virtual depth domain, and I′2 is the corner of I2, corrected by the geometric observations for corners such as I0, I3, and I′1 in the virtual depth domain.

**Optimization of the coordinates of the corner points in the cluster via step-by-step diffusion**. Based on the geometric observation mode for the virtual depth domain, a corner point blur feature quantization strategy based on the dual features of light field light propagation and the image gradient is proposed. Then, the corner point coordinates are refined via multilevel iterative processing according to the quantized blur values, as shown in Figure 8.

(1) Blur feature quantization of corner points based on the dual features of light field ray propagation and the image gradient.

Multiple corner points $\left\{I_{0,\;}I_{1,}\ldots ,I_{j}\right\}$ within the image-space cluster correspond to a unique corner point O on the black and white checkerboard in the object space, and according to the thin lens imaging principle, the blur degree of $\left\{I_{0,}{\;I}_{1,}\ldots ,I_{j}\right\}$ is related only to the type of MLA. Based on geometric optics and image gradient information, the degree of blur of corner $\left\{I_{0,\;}I_{1,}\ldots ,I_{j}\right\}$ can be quantified as follows:

(11) $$S=\alpha *\left(\frac{\lambda ∆{\bar{c}}_{i}}{2}\cdot v^{-1}+\left(q_{i}^{\prime }\;-\frac{\lambda ∆{\bar{c}}_{i}}{2}\right)\right)+\beta *\left(\frac{1}{n}\cdot \sum_{x}\sum_{y}{S\left(x,y\right)}^{2}\right)\;$$

where S represents the blur score, where the higher the value, the clearer the corner points. The weighting parameters are set as α = 0.4 and β = 0.6, based on empirical evaluation across multiple datasets. The first term, weighted by α, is derived from a geometric optics perspective and reflects the spatial consistency between the virtual projection and the expected corner location, as inspired by Labussière et al. [20]. The second term, weighted by β, measures the visual blur degree of the corner in the micro-image. Since corner detection ultimately relies on image content, the image-based term is assigned a slightly higher weight to reflect its greater reliability in practical feature localization; $∆{\bar{c}}_{i}$ represents the mean diameter of the micro-image of the *i* type; $\lambda =\frac{D}{D+d}$ represents the proportional coefficient between the micro-image and the MLA; v represents the virtual depth; qi′=qi+∆c¯i2, $S\left(x,y\right)=\sqrt{G_{x}*I\left(x,y\right)+G_{y}*I\left(x,y\right)}$ represents the horizontal and vertical gradient value at coordinate (x,y) as calculated by the Sobel operator, and n represents the total number of pixels in the micro-image.

(2) Calculation of the initial virtual depth.

Grading is based on the blur quantization value of the corner points. For each corner point cluster, the k-means method is applied to divide the corner point set $P=\{I_{0,\;}{\;I}_{1,\;}\ldots ,I_{j}\}$ into multiple corner point subsets with different blur levels according to the fuzziness score. Assuming that there are three types of MLA, the corner point set can be divided into three mutually exclusive subsets: $P_{A}=\left\{I_{i}\in P\right|\;S(I_{i})=A\}$, $P_{B}=\left\{I_{i}\in P\right|\;S(I_{i})=B\}$, and $P_{C}=\left\{I_{i}\in P\right|\;S(I_{i})=C\}$, where *A*, *B*, and *C* represent the three levels of clarity, sub-clarity, and blur, respectively.

To avoid the accuracy loss caused by MLA distortion and vignetting effects, we select at least three corner points close to the center of the micro-image from the corner point set $P_{A}$ of the clarity level and use the virtual depth geometric relationship (Formula (9)) to perform pairwise forward intersection to calculate the virtual depth v. The average is taken as the initial virtual depth value of the cluster, that is, the position of the virtual image point, as shown in $P_{v}$.

(3) Optimization of the corner point coordinates via a diffusion method.

By using each corner point $I_{i}$ in the set of clear corner points $P_{A}$ in the cluster, the virtual image point $P_{v}$ and Formula (10) form the rear intersection. The virtual image point $P_{v}$ is back-projected into the micro-image, where the target corner point $I_{j}$ is in the secondary definition corner point $P_{B}$, and the median is considered to be the optimized corner point coordinate.

To avoid errors, the pixel blocks around the corner points are fitted, and the local maximum is used to represent the refined corner point coordinates. This optimization process is applied to all corner points in $P_{B}$. Then, the clear corner point set $P_{A}$ and the optimized sub-clear corner point set $P_{B}$ are used as inputs, and more than six corner points close to the center of the micro-image are selected. Finally, the new virtual depth value is calculated and averaged to obtain the virtual depth $v^{\prime }$ of the new cluster. On the basis of the clear corner point set $P_{A}$ and the updated sub-clear corner point set $P_{B}$, the same optimization strategy is adopted to optimize the coordinates of the corner points in the fuzzy corner point set $P_{C}$.

### 3.3. Nonlinear Optimization Strategy with Hierarchical Decoupling

Nonlinear optimization is the last step in camera calibration. Its function is to minimize the reprojection error or maximize a certain likelihood function by adjusting the initially estimated intrinsic and extrinsic parameters of the camera, light direction, scene depth, and other information to obtain accurate light restoration results. The existing methods usually use nonlinear optimization algorithms to optimize all of the parameters at once; however, this single-process optimization of all parameters may be sensitive to the initial values, resulting in an inability to converge to the optimal solution.

**Figure 9:**
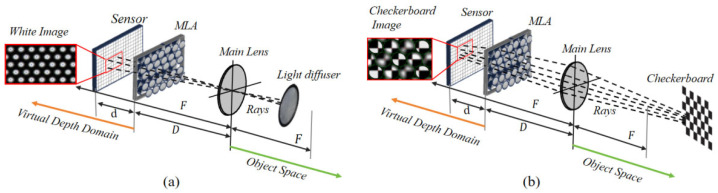
Light beams reconstructed by a light field camera. (**a**) represents the light beam reconstructed from the center of the micro-image and (**b**) represents the light beam reconstructed from the corner features.

The intrinsic structure of the plenoptic camera 2.0 is composed of a main lens, an MLA, and a sensor element. In a scenario in which a white image and a black and white checkerboard are used as calibration objects, this structure enables the light field camera to capture two types of light: central light (as shown in Figure 9a) and the light of the corner feature (as shown in Figure 9b). Following the principle of “simplifying the beam solution problem and avoiding error accumulation”, we use these two types of light to construct the beam equation, introduce the progressive idea of multilayer grading, and propose a decoupled stacked nonlinear optimization, which primarily includes the construction of the projection model, the optimization of the MLA parameters assisted by the central light, the initial estimation and optimization of the extrinsic parameters, and the construction of the global nonlinear optimization function, which accounts for the credibility weight.

**Construction of the projection model**. To make better use of the feature that micro-images with different blurs are represented by MLAs with different focal lengths, the feature points in the micro-image space are represented by the center and radius, that is, $p={\;[u,v,\rho ,1]}^{T}$, and follow the projection model proposed by Labussière et al. [20]., as defined in Formula (12).

(12) $$\left[\frac{\frac{u}{v}}{\frac{\rho }{1}}\right]\propto P(i,k,l)\cdot T_{u}(k,l)\cdot \emptyset (K(F)\cdot T_{c}\cdot p_{w})\;$$

where P(i,k,l) represents the projection matrix of the MLA numbered (k,l) of type *i*, which accounts for the degree of blur. $T_{u}(k,l)$ represents the pose of MLA $\left(k,l\right)$ expressed in the camera coordinate system, the function ∅(·) models the lateral distortion, $T_{c}$ is the pose of the main lens relative to the world coordinate system, and $p_{w}={\;[x,y,z,1]}^{T}$ represents the three-dimensional point coordinates of the real world. The intrinsic parameters of the light field camera include 16 + *i* parameters, including the focal length F of the main lens and its five lateral distortion coefficients $Q_{1}$, $Q_{2}$, $Q_{3}$, $P_{1}$, and ${\;P}_{2}$; the sensor translations d and ($u_{0},\;v_{0}$); the pose of the MLA, including three rotations $\theta_{x}$, $\theta_{y}$, and $\theta_{z}$; three translations $t_{x}$, $t_{y}$, and D; the spacing of the MLA ∆μ; and the focal length of the *i* MLA $f\left(i\right),\;i=0,1,2\ldots$.

**Center-ray-assisted MLA parameter optimization**. By using the extracted high-confidence center point, the initially estimated MLA center and the main lens center, the light reflected by the light diffuser is inversely constructed, as shown by the dotted line in Figure 9a. In other words, a center ray can be regarded to emit from the light diffuser, passing through the center of the main lens and then passing through the center of MLA (*k*, *l*) to reach the sensor element for imaging. Based on this type of light, a center ray projection model is established to calculate the cost function of the observed value and the reprojected value.

(13) $$\Theta (MI)=\sum {\left\|C_{k,l}-\prod_{k,l}\left(p_{w}^{0}\right)\right\|}^{2}$$

where MI represents the set of parameters related to the MLAs that need to be optimized, $C_{k,l}$ represents the center of the micro-image, and $p_{w}^{0}$ represents the object point on the light diffuse reflector in the real world.

The Levenberg–Marquardt algorithm is used for iterative optimization to obtain the optimized values of the parameters related to the MLA. MI includes the distance from the MLA to the main lens (*D*), the distance from the MLA to the sensor (*d*), and the rotation and translation of the MLA relative to the main lens (θ,t).

**Initial estimation and optimization of the extrinsic parameters**. For each corner point cluster, the weight of the corner point is calculated according to the blur radius, and the weighted average center of gravity is calculated. The center of gravity of the corner point cluster can be regarded as the image point of the object-point projected through the pinhole model. For each frame, the intrinsic parameters are fixed, and the initial extrinsic parameters are estimated via the P3P method. A corner point projection model is established on the basis of the intrinsic parameters and initial extrinsic parameters of the MLA parameter optimization assisted by the center ray. The intrinsic parameters are still fixed, and the cost function is constructed by using the clearest corner points to optimize the extrinsic parameters.

(14) $$\Theta (E)=\sum {\left\|p_{k,l}^{n}-\prod_{k,l}\left(p_{w}^{n}\right)\right\|}^{2}$$

where E represents the set of extrinsic parameters to be optimized, $p_{k,l}^{n}$ represents the coordinates of the corner points detected in the *n* frame image, and $p_{w}^{n}$ represents the coordinates of the checkerboard corner points in the real world.

**Corner point removal and blur radius optimization**. After the optimization is complete, the object point is reprojected into the micro-image, and the reprojection error of each corner point is calculated. If the reprojection error of the corner point is greater than a certain threshold (generally twice the root mean square value of the reprojection error in the previous step), the corner point is directly removed; otherwise, it is retained. According to Formulas (3) and (9), the blur radius r is related to v, *D*, and *d*. When these values change after central ray-assisted MLA parameter optimization and the initial estimation and optimization steps for the extrinsic parameters, the blur radius can be recalculated for the corner points that meet the threshold to solve for the intrinsic and extrinsic parameters of the camera with higher accuracy during global nonlinear optimization.

**Global nonlinear optimization**. Finally, we propose a new cost function for global nonlinear optimization. Both the intrinsic and extrinsic parameters are optimized in one iteration. The new cost function is composed of the corner reprojection error, blur radius reprojection error, and micro-image center reprojection error.

(15) $$\Theta (T)=\sum {\left\|(p_{k,l}^{n}-\prod_{k,l}(p_{w}^{n}))\cdot w_{k,l}^{n}\right\|}^{2}+\sum {\left\|r_{k,l}^{n}-\prod_{k,l}\left(p_{w}^{n}\right)\right\|}^{2}+\Theta (MI)$$

where *T* represents the set of all intrinsic and extrinsic parameters to be optimized, $\sum {\left\|(p_{k,l}^{n}-\prod_{k,l}(p_{w}^{n}))\cdot w_{k,l}^{n}\right\|}^{2}$ represents the corner cost function considering the blur perception characteristics, and $w_{k,l}^{n}$ refers to the normalized weight corresponding to the quantized blur degree of the corner point. The weighting function is as follows:

(16) $$w_{k,l}^{n}=\alpha \;+(1-\alpha )\cdot e^{-(r_{k,l}^{n}-min(r_{k,l}^{n}))}\;$$

where $r_{k,l}^{n}$ represents the blur radius in the *n* frame image; α is the lowest weight value, which is generally set to 0.4. This value was empirically determined based on a series of calibration experiments conducted on multiple focused light field camera models. It provides a good trade-off between sensitivity to blur variations and robustness against noise. This threshold can be adjusted according to specific hardware configurations or imaging conditions; and $min(r_{k,l}^{n})$ represents the minimum value of the blur radius in the *n* frame.

## 4. Experiments and Analysis

### 4.1. Experiment Setup

**Figure 10:**
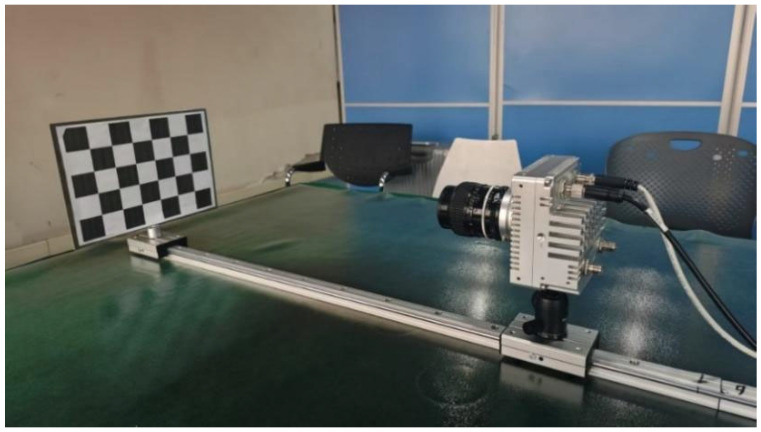
Original checkerboard image acquired with fixed step length.

To validate the effectiveness of our proposed method, we evaluated it in a controlled environment using both real and simulated data acquired with a multi-focus plenoptic camera. Our experimental setup is shown in Figure 10. The camera is mounted on a linear motion stage with micron-level precision. The target plane is orthogonal to the translation axis, and the camera optical axis is aligned with this axis. Table 1 lists the approximate absolute distances for images captured with corresponding step sizes.

1. Hardware Environment
  The light field cameras used in the experiments primarily included two multifocal plenoptic cameras, namely, the Raytrix R12 and HR260. The Raytrix R12 is produced by the German company Raytrix, and the HR260 is independently developed by our research group. The detailed specifications of the three light field cameras are as follows:
  (1) The main lens used by Raytrix R12 is a Nikon AF Nikkor F/1.8D with a focal length of 50 mm. The MLA consists of 176 × 152 microlenses with three focal lengths arranged crosswise. The sensor is a Basler boA4000-KC with a pixel size of 0.0055 mm, a resolution of 4080 × 3068, and a working distance of approximately 0.1~5 m.
  (2) The main lens for the HR260 has a focal length of 105 mm, and the MLA consists of 65 × 50 microlenses with three focal lengths arranged crosswise. The sensor pixel size is 0.0037 mm, the resolution is 6240 × 4168, and the working distance is approximately 0.1~100 m.
2. Software Environment

When capturing light field images, we set the shutter speed to 5 ms. When capturing white images, we set the gain to maximum. For the Raytrix data, we used their proprietary software, RxLive (v4.0.50.2), to calibrate the camera and calculate the depth maps used in the evaluation. For the HR260 data, we used our own imaging software to generate the light field images.

These data include publicly available datasets acquired with the Raytrix R12 camera by Labussière et al. [20,21], alongside self-collected datasets from HR260 cameras. Specifically, the sequences *R12-A*~*R12-C* and *HR260-A*~*HR260-B* correspond to these respective devices. To comprehensively validate the accuracy of the calibrated intrinsic and extrinsic parameters, the datasets cover multi-view and controlled translational sequences captured at a fixed focus. A detailed introduction to the dataset is provided in Table 1.

3. Datasets
  We establish three datasets with different focus distances ***H***, obtained from two different light field cameras, with detailed parameter settings as shown in Table 1. Each dataset consists of the following:
  (1) White raw plenoptic images captured at different apertures ($N\in \left\{5.66,8.0,11.31,16.0\right\}$) using a light diffuser mounted on the main objective lens.
  (2) Target images captured at different poses (distances and directions), divided into two subsets, one for the calibration process (16 images) and the other for reprojection error evaluation (15 images).
  (3) White raw plenoptic images acquired under the same illumination conditions and the same aperture are used for calibration target acquisition for dehalation, as well as calibration targets acquired through controlled translation motion for quantitative evaluation.

**Figure 11:**
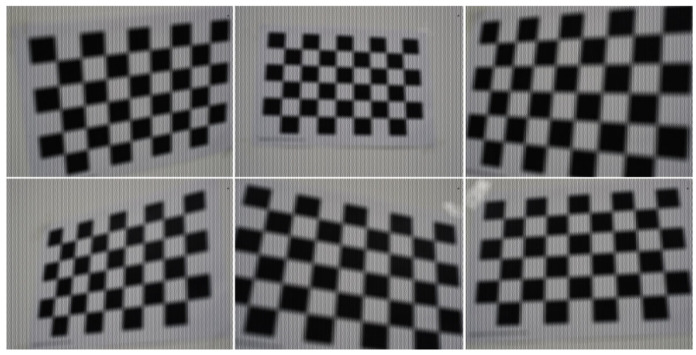
Checkerboard images captured by the Raytrix R12.

Figure 11 shows some of the captured light field images, and Table 1 gives the detailed parameter settings for each dataset. Our dataset can be downloaded from public repository: https://github.com/LightFieldVision/LightField2.0-datasets-for-calibration, accessed on 6 August 2025.

4. Simulation Environment

**Figure 12:**
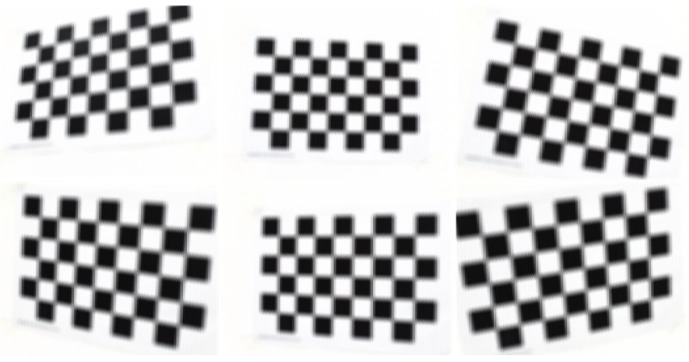
The simulation light field checkerboard images.

Based on the method proposed in reference [30], three datasets were generated using its open source code. These datasets provide ground truth corner positions, enabling direct accuracy assessment. The camera parameters are set as follows: the MLA structure is arranged in a hexagonal shape and consists of three types microlenses. It should be noted that ***F*** represents the focal length of the main lens in millimeters, ***F*** is set to 105 mm, and $s_{xy}$ represents the pixel size of the sensor in micrometers where $s_{xy}$ is set to 5.5 µm. The detailed simulation parameters are listed in Table 2, and Figure 12 shows some of the simulation light field images

### 4.2. Ablation Study

To evaluate the effectiveness and performance of the proposed method, we conducted a comprehensive ablation study. This study systematically isolates and assesses the contributions of three key modules in our approach: corner feature extraction, principal ray-assisted optimization of microlens array parameters, and nonlinear optimization. The goal is to evaluate the individual impact of each module on the overall calibration performance and to gain a deeper understanding of their respective roles.

#### 4.2.1. Evaluation Metrics

We use the reprojection error as a key metric to evaluate performance.

Mean Reprojection Error: This metric is a critical and commonly used error evaluation criterion in pose estimation and 3D reconstruction. It can also be used to determine whether the pose estimation has converged. It represents the average distance, measured in pixels, between the reprojected position of all 3D points onto the images and their corresponding observed positions in the actual images across all matched points. The formula is as follows:

(17) $$MSE=\frac{1}{M}\sum_{j=1}^{M}{\left\|p_{j}-p_{j}^{\prime }\right\|}_{2}$$

Here, M denotes the number of observations for each 3D point; $p_{j}={\left[u_{j},v_{j}\right]}^{T}$ represents the ground truth image point of the j-th observation; and $p_{j}^{\prime }={\left[u_{j}^{\prime },v_{j}^{\prime }\right]}^{T}$ denotes the reprojected point of the corresponding 3D point onto the same image.

#### 4.2.2. Experimental Setup

To evaluate the contribution of each key module, we conducted ablation experiments using the *R12-A*, *HR260-A*, and *HR260-B* datasets described in Section 4.1. The specific experimental configurations are as follows:

(1) Without neighboring blur-aware corner feature extraction: This configuration evaluates the system’s performance across different datasets when the blur-aware corner feature extraction guided by neighboring views is disabled.
(2) Without principal ray-assisted microlens array parameter optimization: This setting assesses the system’s accuracy on various datasets when the optimization of microlens array parameters using principal rays is not employed.
(3) Without decoupled hierarchical nonlinear optimization: This test examines the system’s accuracy on different datasets when the decoupled hierarchical nonlinear optimization module is omitted.
(4) Full system: This configuration includes all modules, namely the neighboring blur-aware corner feature extraction, principal ray-assisted microlens array parameter optimization, and decoupled hierarchical nonlinear optimization.

#### 4.2.3. Ablation Results and Contribution Impact

Table 3 summarizes the calibration accuracy under different module configurations. The average corner reprojection error (RMSE) across three datasets—R12-A, HR260-A, and HR260-B—was used as the evaluation metric. The following observations can be drawn:

(1) Effect of blur-aware corner extraction

When the blur-aware corner feature extraction is disabled, the reprojection error increases significantly on all datasets. For instance, the RMSE on HR260-B increases from 1.250 pixels (full system) to 1.945 pixels. This confirms that the proposed corner refinement strategy—guided by virtual depth and blur quantization—is effective in improving corner localization accuracy.

(2) Effect of center-ray-assisted MLA optimization

Disabling the center-ray-based optimization leads to a moderate accuracy drop on R12-A (from 0.527 to 0.610 pixels) and HR260-A (from 0.572 to 1.171 pixels), but a drastic drop on HR260-B (from 1.250 to 14.421 pixels). This indicates that the optimization of microlens geometry using center rays is particularly important in scenes with long-range depth variation or strong blur, such as HR260-B.

(3) Effect of decoupled nonlinear optimization:

Removing the staged nonlinear optimization leads to a consistent performance drop, especially in the HR260-B dataset, where RMSE increases to 17.125 pixels. This suggests that direct joint optimization without prior parameter refinement may suffer from poor convergence in complex configurations.

(4) Overall effectiveness:

The full system consistently achieves the lowest reprojection errors across all datasets. This demonstrates that each of the proposed modules contributes to improved calibration accuracy and that their integration produces the most stable and accurate results.

These results validate the importance of each innovation in our pipeline and highlight their complementary roles in enhancing calibration robustness and precision.

### 4.3. Corner Feature Extraction Accuracy Evaluation

The quality of corner feature extraction from micro-images with different blur levels in multifocal focused light field images is one of the main problems addressed in this paper. To verify the effectiveness of the proposed method in terms of the feature corner accuracy, we used three sets of light field simulation image datasets to quantitatively compare and analyze the proposed method with classic methods such as those from Nousias [14] and Noury [15]. The evaluation indicators include the mean error (Mean) and standard deviation (Std).

Table 4 lists the performance of the extracted corner points and the true corner points in terms of the error mean, error standard deviation, maximum error, minimum error, and other indicators on the *R*_1_~*R*_3_ datasets. The following conclusions can be drawn from the experimental results:

(1) As the image blur changes, the Nousias method and Noury method exhibit large fluctuations. Furthermore, the error gradually increases with increasing blur, indicating that the accuracy of these two methods is strongly affected by blur, which also verifies the problem raised in this paper. However, the proposed method performs smoothly, and the accuracies of the three types of corner points are almost the same.
(2) Noury’s method and the proposed method have multiple identical numerical values in terms of the minimum error because the first step of the corner feature extraction strategy proposed in this paper, which is guided by neighboring blur information, uses the template matching algorithm introduced by Noury et al. [15] to detect the corner coordinates of the micro-image. The corner coordinates with the minimum error are the detection results corresponding to the clearest corners. The corner feature extraction strategy uses the coordinates of these clearest corners to construct geometric observations in the virtual depth domain to optimize the coordinates of other corners.
(3) The overall performance of the proposed method is the best in terms of the two indicators of the error mean and standard deviation, as the experimental results for the proposed method are the smallest. Compared with the second-ranked method, the accuracy is improved by 11%, 57%, 60%, 44%, and 43%, respectively, with an average improvement of more than 30%. These improved results can be obtained due to the corner feature extraction strategy guided by neighboring blur information proposed in this paper. This approach uses the geometric relationship between the epipolar lines between micro-images and the virtual depth space to construct a multi constraint model and then, on this basis, constructs a geometric observation model of the virtual depth domain. It next quantifies and grades the degree of blur imaging for multiple corner points in the cluster, in turn iteratively filtering the noise in the cluster according to the step-by-step diffusion method and optimizes the coordinates of the corner points from clear to blur. Therefore, the proposed method refines relatively blurred corner coordinates and effectively improves the extraction quality of corner features.

**Figure 13:**
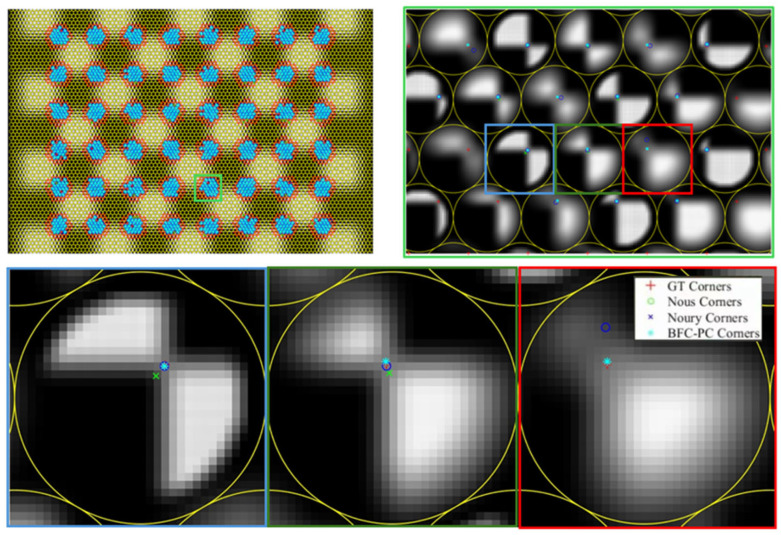
Corner point coordinates extracted via different methods and the true corner point coordinates. The true values of the corner point coordinates and the corner point coordinates extracted via the Nousias method, the Noury method, and the proposed method are marked with different symbols. In addition, there are four types of corner point coordinates in *R*_2_.

To more intuitively show the error between the extracted corner point coordinates and the real corner point coordinates, we randomly selected local areas in the two groups of simulated light field images R2 for detailed display, as shown in Figure 13. The figure shows that the corner point coordinates extracted by the proposed method are basically the closest to the true corner point coordinates, whether in clear or blurred micro-images.

### 4.4. Accuracy Assessment of the Intrinsic Calibration Parameters

Existing methods usually use the reprojection error (RMSE) as the main indicator to measure the accuracy of the intrinsic calibration parameters. We selected five public datasets and field-collected datasets, including *R12-A*~*R12-C* and *HR260-A*~*HR260-B*, as experimental data and quantitatively compared and analyzed the proposed method with well-known methods such as those from Labussière [20], Nousias [14], and Liu [19]. The light field image data used in the experiment were all taken by a multifocal focused light field camera composed of three different types of microlenses.

The reprojection errors of each method are shown in Table 5, and the estimated intrinsic parameters of the camera can be found in Table 6, Table 7, Table 8, Table 9 and Table 10. The following conclusions can be drawn from the experimental results:

(1) When the given camera parameters are limited, only the focal length of the main lens is considered. Although the reprojection errors of the Nousias and Liu methods are different, especially the Liu method, which has a better reprojection error performance, the estimated focal length of the main lens is significantly different from the given value, so the accuracy of the estimated intrinsic parameters is questionable.
(2) The focal length of the main lens estimated by the proposed method and the Labussière method is closest to the given value, and the reprojection error of the proposed method is the smallest at approximately 0.5 pixels; however, the reprojection error of the Labussière method can reach 2.080 pixels. Therefore, the proposed method has the best comprehensive performance in terms of calibrating the intrinsic parameters because the proposed method constructs the quantification and classification rules related to the micro-image clarity to account for the blur-perceived full-light characteristics and uses the geometric observation mode of the virtual depth domain to effectively improve the extraction quality for corner features.
(3) Compared with the given main lens focal length parameters, the main lens focal length values estimated by the Nousias and Liu methods are still significantly different from the given values, and the reprojection error is large, even reaching 6 pixels. The main lens focal length values estimated by the Labussière method are all close to the given values, but the reprojection error, an indicator that characterizes the accuracy of the intrinsic parameters, is too large, with a maximum of 20.049 pixels. Therefore, the robust performance of the proposed method is the best because it introduces blur-aware plenoptic features, constructs a step-by-step diffusion corner point optimization model to achieve high-quality extraction of corner point coordinates, and establishes a decoupled stacked intrinsic and extrinsic parameter optimization strategy to ensure the accuracy and robustness of the calibration.

### 4.5. Comparative Analysis of the Z-Axis Translation Error

To further validate the effectiveness of the calibration parameters, the *Z*-axis displacement error index was employed to assess the accuracy of the extrinsic parameters. This approach is similar to those used by Labussière [20], Nousias [14], and Liu [19]. A total of 6 datasets, comprising both public and field-collected data, were selected and three different cameras were used: the Raytrix R12 and HR260. The datasets were acquired on a controlled acquisition platform, where the translation distance was precisely managed.

We compared the estimated displacement of the extrinsic parameter along the *Z*-axis with the ground truth displacement value to calculate the translation error. The corresponding calculation formula is as follows:

(18) $$\varepsilon_{z}\;(\delta_{z})=\eta^{-1}\;\sum_{(T_{i},T_{j})|z_{i}-z_{j}=\delta_{z}}\left|\delta_{z}\;-\hat{\delta_{z}}\right|/\delta_{z}\;$$

In this context, $\varepsilon_{z}$ denotes the translation error, while $\delta_{z}$ signifies the ground truth displacement. η is the normalization constantly associated with the number of frame pairs; $\left(T_{i},T_{j}\right)$ represents a frame pair, with indices *i* and *j* indicating the specific frame numbers; $z_{i}$ and $z_{j}$ correspond to the ground truth distance values for the two images within the frame pair; and $\hat{\delta_{z}}$ represents the estimated displacement value, $\hat{\delta_{z}}=\hat{z_{i}}-\hat{z_{j}}$. Note: Equation (17) and the associated index notation are directly adopted from the original definition of the baseline method, Labussière [20], in order to ensure consistency in implementation and comparison.

We used four methods, namely, the BFC-PC, Labussière, Nousias, and Liu, to calibrate 5 sets of data from three different types of cameras and calculated the extrinsic parameter errors between frame pairs in addition to the error mean and standard deviation. For each camera model, a representative set of extrinsic parameter error distributions was randomly selected, along with a comprehensive error bar chart illustrating the overall calibration performance.

**Figure 14:**
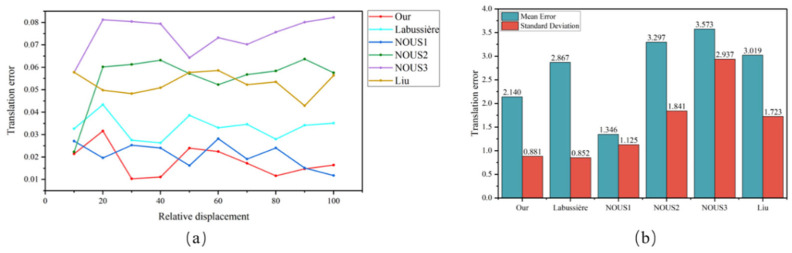
Statistics of the extrinsic parameter errors produced by the Raytrix R12 camera. (**a**) presents the extrinsic parameter errors under 10 different displacements in R12-A and (**b**) depicts the means and standard deviations of the extrinsic parameter errors for R12-A, R12-B, and R12-C.

**Figure 15:**
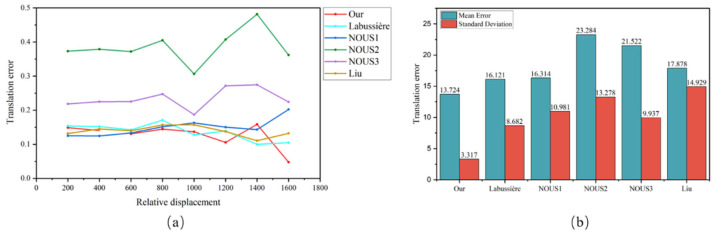
Statistics of the extrinsic parameter errors for the HR260 camera. (**a**) presents the extrinsic parameter errors under 8 different displacements in HR260-A and (**b**) depicts the means and standard deviations of the extrinsic parameter errors for HR260-A and HR260-B.

Figure 14 and Figure 15 show the statistical results of the extrinsic parameter errors for the three cameras. Figure 14a and Figure 15a present the statistics of the extrinsic parameter errors between frame pairs under different displacement conditions contained in the dataset, while Figure 14b and Figure 15b present the means and standard deviations of the extrinsic parameter errors. The bar graph represents the mean, and the straight-line segment represents the standard deviation. The following conclusions can be made:

(1) Comparative Performance in terms of the Extrinsic Parameter Error

The proposed method demonstrates superior performance in terms of the extrinsic parameter error across all three camera models. Specifically, on the *R12-A* dataset (Figure 14a), our method achieves an optimal extrinsic parameter error for 7 out of 10 datasets, with the remaining 3 datasets resulting in marginally greater errors than the Nousias method. Notably, the Nousias method exhibits significant performance variability across different microlens types. Furthermore, on the *HR260-A* dataset (Figure 15a), while all four methods show substantial error fluctuations, our method maintains superior stability, with error values ranging between 4.77% and 15.85%; these values are significantly lower than those of the competing methods. These results collectively demonstrate the robustness of our method across varying intrinsic parameter configurations in different light field cameras.

(2) Consistency Analysis via the Standard Deviation

The standard deviation serves as a critical metric for evaluating calibration consistency across datasets and method generalizability to different camera configurations. As evidenced by the error bar graphs in Figure 14b and Figure 15b, our method consistently results in the smallest standard deviation, indicating minimal performance variation across datasets. This consistency not only reflects the reliability of our calibration method but also confirms the accuracy of the intrinsic parameter estimation. Consequently, our method demonstrates both accuracy and consistency when applied to diverse camera configurations.

(3) Distance-dependent Performance Analysis

The proposed method maintains stable performance across varying shooting distances, with no observable correlation between the extrinsic parameter error and an increased shooting distance. This distance-independent performance enables reliable pose readjustment and precise estimation regardless of the shooting conditions.

### 4.6. Runtime Efficiency Comparison

To further evaluate the practicality of the proposed method, we measured the runtime of each stage in our calibration pipeline on four representative datasets (*R12-A*, *R12-B*, *HR260-A*, and *HR260-B*) and compared the results with those of the method proposed by Labussière et al. [20]. Since the methods by NOUS [14] and Liu [19] are implemented in MATLAB (MATLAB 2020b), and our method and Labussière’s are implemented in C++ (C++ 11), a direct runtime comparison across different programming languages would be misleading. Therefore, we excluded NOUS and Liu from the runtime comparison.

The runtime statistics are summarized in Table 11. Despite incorporating several additional modules (e.g., center-ray-assisted microlens array optimization and blur-aware corner refinement), the overall computational time of our method is comparable to that of Labussière’s method, and in some cases, superior—particularly in the global nonlinear optimization stage. This improvement stems from better parameter initialization and the cascaded optimization structure, which accelerates convergence.

Moreover, the newly introduced modules are computationally lightweight. The center ray optimization and extrinsic parameter refinement typically require less than 20 s per dataset, which demonstrates that the performance gains do not come at the cost of significantly increased runtime. These results indicate that the proposed method offers a favorable trade-off between accuracy and efficiency and is scalable for high-resolution plenoptic datasets.

**Figure 16:**
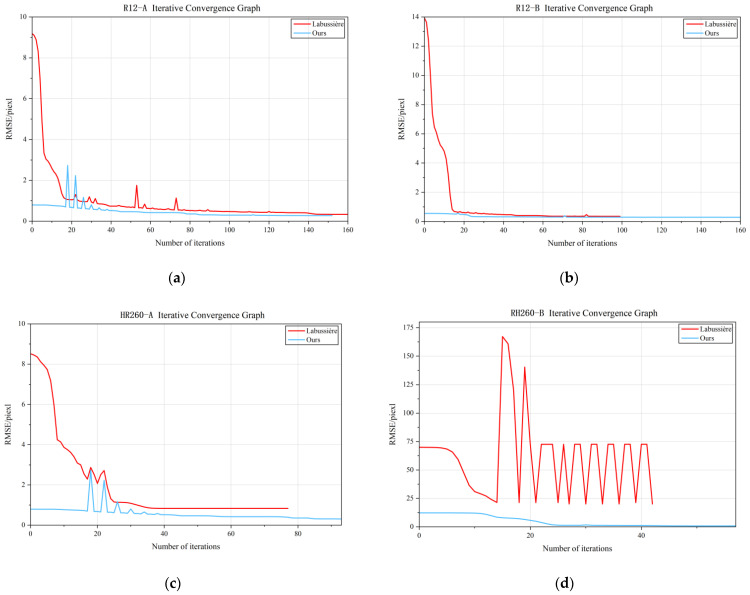
Iterative Convergence Graph. (**a**) represents the number of iterations and convergence trend of *R12-A*; (**b**) represents the number of iterations and convergence trend of *R12-B*; (**c**) represents the number of iterations and convergence trend of *HR260-A*; (**d**) represents the number of iterations and convergence trend of *HR260-B*.

In addition, the convergence trends of different optimization stages are visualized in Figure 16. The figure illustrates the reduction in the reprojection error over iterations for both the decoupled nonlinear optimization and global optimization stages. It can be observed that the proposed method exhibits stable convergence behavior, with rapid error reduction in the early iterations and smooth convergence in later stages. The decoupled structure allows intermediate parameters to be progressively refined, which helps improve the convergence rate and stability of the subsequent global optimization. These curves further confirm the effectiveness of the cascaded design in accelerating convergence while maintaining robustness.

## 5. Conclusions

We propose a blur feature-guided cascaded calibration method for plenoptic cameras. The blur-aware plenoptic feature is introduced to construct a step-by-step diffusion corner optimization model and a decoupled cascade intrinsic and extrinsic parameter optimization strategy to achieve high-quality extraction of corner coordinates and robust calibration. First, high-precision center point extraction and initial estimation of intrinsic parameters are performed, and then corner feature extraction guided by neighboring blur information is performed. Based on the Fermat principle and Gaussian imaging principle, a geometric observation model of the virtual depth domain unique to the plenoptic camera is constructed; then, a decoupled cascade nonlinear optimization strategy is proposed. Experimental verification and analysis were conducted by using public datasets and captured datasets. The results reveal that the proposed method excels in corner feature extraction, calibration accuracy of both intrinsic and extrinsic parameters, and calibration sensitivity when applied to multifocal focused plenoptic cameras, highlighting its advancement and robustness.

Moreover, despite the multi-stage optimization design, the proposed method maintains high computational efficiency, striking a favorable balance between accuracy and runtime cost, and proving its practical applicability to real-world engineering scenarios.

While the proposed method demonstrates high accuracy and robustness in the calibration of multifocal plenoptic cameras, several limitations still exist. Currently, the method is designed for offline calibration scenarios and has not yet been extended to real-time or online environments. Additionally, the performance of the blur-aware corner extraction may be affected under extremely low-texture or high-noise conditions, where blur estimation becomes less stable. Future work will focus on enhancing the method’s adaptability to online applications and improving robustness under challenging imaging conditions.

## Figures and Tables

**Table 1:** Raw image datasets collected by the three light field cameras. ***H*** refers to the focus distance, where all distances are in millimeters; Scale refers to the square size of the checkerboard; and Depth Distance refers to the setting of the linear motion workbench.

Dataset	*H* (mm)	Checkerboard		Depth Distance		
		Size (Rows × Cols)	Scale (mm)	Min (mm)	Max (mm)	Step (mm)
*R12-A*	450	9 × 5	10	265	385	10
*R12-B*	1000	8 × 5	20	450	900	50
*R12-C*	∞	6 × 4	30	400	1250	50
*HR260-A*	1500	7 × 5	35	1200	2200	100
*HR260-B*	4000	6 × 4	40	1000	2600	200

**Table 2:** Simulated light field image dataset. Resolution represents the resolution of the image; $D_{mi}$ represents the radius of the micro-image in pixels; ***D*** represents the distance from the main lens to the MLA in millimeters; ***d*** represents the distance from the MLA to the sensor in millimeters; and $f_{i}$ represents the focal length of the different types of MLAs contained in the MLA, where ***i*** = 0, 1, 2, in millimeters.

Dataset	Resolution	$D_{mi}$ (Pixels)	*D* (mm)	*D* (mm)	$f_{i}$ (mm)
*R*_1_	2000 × 3000	23.4	101.43	1.32	1.35/1.62/1.95
*R*_2_	2000 × 3000	60	99.62	1.32	1.34/1.75/2.25
*R*_3_	6000 × 8000	130	99.41	1.30	1.35/1.75/2.25

**Table 3:** Ablation study results.

Configuration	RMSE (Pixel)		
	*R12-A*	*HR260-A*	*HR260-B*
Without neighboring blur-aware corner extraction	0.936	1.541	1.945
Without center-ray-assisted microlens array optimization	0.610	1.171	14.421
Without decoupled nonlinear optimization	0.686	1.235	17.125
Full system (all modules included)	0.527	0.572	1.250

**Table 4:** Statistics of the pixel errors between the extracted corner points and the true values. The mean error (mean), standard deviation error (std), maximum error (max), and minimum error (min) are shown in turn. Type_1_ represents the corner points in the clearest image; Type_2_ represents the corner points in the second-clearest image; Type_3_ represents the corner points in the most blurred image; and All represents the corner points in the three types of images.

Dataset	Method	Type_1_	Type_2_	Type_3_	All
*R*_1_	Nousias	(0.83, 0.42) (2.28, 0.04)	(0.79, 0.41) (2.22, 0.02)	(0.95, 0.45) (2.56, 0.10)	(0.84,0.43) (2.56,0.02)
	Noury	(0.81, 0.37) (2.78, 0.03)	(0.82, 0.37) (2.06, 0.01)	(0.90, 0.47) (2.87, 0.03)	(0.84, 0.40) (2.87, 0.01)
	BFC-PC	(0.81, 0.37) (2.78, 0.03)	(0.71, 0.24) (1.04, 0.07)	(0.71, 0.23) (1.24, 0.03)	(0.74, 0.29) (2.78, 0.03)
*R*_2_	Nousias	(1.37, 0.69) (3.22, 0.13)	(1.55, 0.76) (3.63, 0.16)	(2.32, 0.88) (3.89, 0.41)	(1.53, 0.78) (3.89, 0.13)
	Noury	(0.51, 0.33) (2.18, 0.03)	(0.73, 0.39) (2.37, 0.03)	(2.25, 0.80) (2.99, 0.11)	(0.73, 0.59) (2.99, 0.02)
	BFC-PC	(0.51, 0.33) (1.39, 0.03)	(0.43, 0.23) (1.12, 0.03)	(0.43, 0.23) (1.35, 0.01)	(0.46, 0.27) (2.18, 0.01)
*R*_3_	Nousias	(1.82, 0.92) (3.93, 0.08)	(2.91, 1.38) (6.26, 0.88)	(3.17, 1.54) (6.01, 0.23)	(2.43, 1.38) (6.26, 0.08)
	Noury	(1.16, 0.63) (3.15, 0.04)	(2.17, 1.43) (4.83, 0.28)	(2.72, 1.64) (4.95, 0.06)	(1.71, 1.31) (4.95, 0.04)
	BFC-PC	(1.16, 0.63) (3.01, 0.04)	(1.11, 0.61) (3.48, 0.06)	(1.16, 0.62) (2.77, 0.10)	(1.14, 0.63) (3.48, 0.04)

**Table 5:** Statistics of the corner reprojection error (RMSE) in the pixels. In addition to the reprojection error for the corner points, the results for our method and the Labussière method also include the reprojection error of the blur radius, which is the value in parentheses. Underlined values indicate larger reprojection errors.

Datasets	BFC-PC	Labussière	NOUS1	NOUS2	NOUS3	Liu
*R12-A*	0.527 (0.080)	0.856 (0.083)	0.773	0.667	0.958	0.691
*R12-B*	0.421 (0.086)	0.674 (0.183)	0.538	0.519	0.593	0.330
*R12-C*	0.336 (0.040)	0.738 (0.041)	1.287	0.681	0.411	0.338
*HR260-A*	0.572 (0.257)	1.827 (0.293)	1.624	1.432	1.316	0.681
*HR260-B*	1.250 (0.371)	20.049 (12.866)	3.296	19.175	3.514	5.849

**Table 6:** Initial internal parameters for the *R12-A* dataset and the optimized parameters obtained via the proposed method, Labussière method, Nousias method (for each microlens type: NOUS1, NOUS2, and NOUS3), and Liu method. The dashes indicate solutions that did not consider the parameters.

	*R12-A* (*F* = 50 mm, *H* = 450 mm)					
	Ours	Labussière	NOUS1	NOUS2	NOUS3	Liu
F **[mm]**	50.471	49.714	61.305	62.476	63.328	58.233
$Q_{1}$ **[$\times 10^{-5}$]**	16.82	24.66	—	—	—	—
${-Q}_{2}$ **[$\times 10^{-6}$** **]**	2.044	2.998	—	—	—	—
$Q_{3}$ **[$\times 10^{-8}$]**	0.717	1.063	—	—	—	—
$P_{1}$ **[$\times 10^{-5}$]**	−6.8	−14.6	—	—	—	—
$-P_{2}$ **[$\times 10^{-5}$]**	0.556	6.340	—	—	—	—
D ** [mm ** **]**	57.520	56.701	71.131	72.541	73.530	66.985
$-t_{x}$ ** [mm ** **]**	10.90	10.97	—	—	—	—
$-t_{y}$ ** [mm ** **]**	8.419	7.887	—	—	—	—
${-\theta }_{x}$ ** [μrad ** **]**	463.1	843. 1	—	—	—	—
$\theta_{y}$ ** [μrad ** **]**	389.2	637.1	—	—	—	—
$\theta_{z}$ ** [μrad ** **]**	29.1	31.5	—	—	—	—
${∆}_{u}$ ** [μm ** **]**	127.46	127.46	—	—	—	—
$f_{1}$ ** [μm ** **]**	766.26	578.18	—	—	—	—
$f_{2}$ ** [μm ** **]**	689.95	505.42	—	—	—	—
$f_{3}$ ** [μm ** **]**	855.03	552.08	—	—	—	—
$u_{0}$ ** [pix ** **]**	2083.9	2070.9	1984.9	2034.5	1973.7	2085.8
$v_{0}$ ** [pix ** **]**	1513.8	1610.9	1482.1	1481.0	1495.2	1590.3
d ** [μm ** **]**	3,36.48	324.77	585.16	527.59	561.93	455.30

**Table 7:** Initial internal parameters for the *R12-B* dataset and the optimized parameters obtained via the proposed method, Labussière method, Nousias method (for each microlens type: NOUS1, NOUS2, and NOUS3), and Liu method. The dashes indicate solutions that did not consider the parameters.

	***R12-B* (*F* = 50 mm, *H* = 450 mm)**					
	Ours	Labussière	NOUS1	NOUS2	NOUS3	Liu
F **[mm]**	50.170	50.047	53.913	52.988	52.977	48.949
$Q_{1}$ **[$\times 10^{-5}$]**	−0.419	2.900	—	—	—	—
${-Q}_{2}$ **[$\times 10^{-6}$** **]**	0.086	0.300	—	—	—	—
$Q_{3}$ **[$\times 10^{-8}$]**	0.036	0.064	—	—	—	—
$P_{1}$ **[$\times 10^{-5}$]**	8.46	14.13	—	—	—	—
$-P_{2}$ **[$\times 10^{-5}$]**	17.655	21.540	—	—	—	—
D ** [mm ** **]**	52.217	52.125	56.062	55.128	55.124	50.830
$-t_{x}$ ** [mm ** **]**	12.03	12.44	—	—	—	—
$-t_{y}$ ** [mm ** **]**	6.447	5.988	—	—	—	—
${-\theta }_{x}$ ** [μrad ** **]**	436.9	607.2	—	—	—	—
$\theta_{y}$ ** [μrad ** **]**	477.5	514.5	—	—	—	—
$\theta_{z}$ ** [μrad ** **]**	38.5	46.0	—	—	—	—
${∆}_{u}$ ** [μm ** **]**	127.46	127.45	—	—	—	—
$f_{1}$ ** [μm ** **]**	708.39	580.49	—	—	—	—
$f_{2}$ ** [μm ** **]**	646.18	504.31	—	—	—	—
$f_{3}$ ** [μm ** **]**	795.08	546.36	—	—	—	—
$u_{0}$ ** [pix ** **]**	1877.2	1958.3	2074.7	2094.7	1837.0	1946.9
$v_{0}$ ** [pix ** **]**	1874.4	1802.9	1640.2	1649.1	1620.4	1668.7
d ** [μm ** **]**	337.83	336.38	447.81	401.93	414.32	324.70

**Table 8:** Initial internal parameters for the *R12-C* dataset and the optimized parameters obtained via the proposed method, Labussière method, Nousias method (for each microlens type: NOUS1, NOUS2, and NOUS3), and Liu method. The dashes indicate solutions that did not consider the parameters.

	*R12-C* (*F* = 50 mm, *H* = ∞)					
	Ours	Labussière	NOUS1	NOUS2	NOUS3	Liu
F **[mm]**	50.197	50.013	51.113	49.919	50.812	44.523
$Q_{1}$ **[$\times 10^{-5}$]**	17.21	18.61	—	—	—	—
${-Q}_{2}$ **[$\times 10^{-6}$** **]**	3.016	2.646	—	—	—	—
$Q_{3}$ **[$\times 10^{-8}$]**	1.406	1.038	—	—	—	—
$P_{1}$ **[$\times 10^{-5}$]**	9.16	19.11	—	—	—	—
$-P_{2}$ **[$\times 10^{-5}$]**	7.245	7.311	—	—	—	—
D ** [mm ** **]**	49.424	49.362	50.331	49.067	49.882	43.819
$-t_{x}$ ** [mm ** **]**	12.76	13.12	—	—	—	—
$-t_{y}$ ** [mm ** **]**	8.153	7.446	—	—	—	—
${-\theta }_{x}$ ** [μrad ** **]**	417.8	490.9	—	—	—	—
$\theta_{y}$ ** [μrad ** **]**	389.7	388.9	—	—	—	—
$\theta_{z}$ ** [μrad ** **]**	35.1	41.1	—	—	—	—
${∆}_{u}$ ** [μm ** **]**	127.46	127.48	—	—	—	—
$f_{1}$ ** [μm ** **]**	650.32	569.88	—	—	—	—
$f_{2}$ ** [μm ** **]**	598.01	491.71	—	—	—	—
$f_{3}$ ** [μm ** **]**	712.68	535.28	—	—	—	—
$u_{0}$ ** [pix ** **]**	1743.7	1692.1	1966.3	1913.8	2052.5	1845.3
$v_{0}$ ** [pix ** **]**	1562.3	1677.8	1484.6	1487.2	1492.7	1514.4
d ** [μm ** **]**	328.63	319.53	357.80	349.99	353.26	274.00

**Table 9:** Initial internal parameters for the *HR260-A* dataset and the optimized parameters obtained via the proposed method, Labussière method, Nousias method (for each microlens type: NOUS1, NOUS2, and NOUS3), and Liu method. The dashes indicate solutions that did not consider the parameters.

	*HR260-A* (*F* = 105 mm, *H* = 1500 mm)					
	Ours	Labussière	NOUS1	NOUS2	NOUS3	Liu
F **[mm]**	105.658	112.129	87.115	52.486	52.227	57.274
$Q_{1}$ **[$\times 10^{-5}$]**	−45.06	11.82	—	—	—	—
${-Q}_{2}$ **[$\times 10^{-6}$** **]**	−14.794	2.066	—	—	—	—
$Q_{3}$ **[$\times 10^{-8}$]**	−9.576	1.143	—	—	—	—
$P_{1}$ **[$\times 10^{-5}$]**	12.08	−2.21	—	—	—	—
$-P_{2}$ **[$\times 10^{-5}$]**	−24.320	6.325	—	—	—	—
D ** [mm ** **]**	109.929	109.086	89.097	50.416	49.497	54.867
$-t_{x}$ ** [mm ** **]**	11.36	10.92	—	—	—	—
$-t_{y}$ ** [mm ** **]**	7.690	7.206	—	—	—	—
${-\theta }_{x}$ ** [μrad ** **]**	352.7	834.6	—	—	—	—
$\theta_{y}$ ** [μrad ** **]**	4752.3	3274.3	—	—	—	—
$\theta_{z}$ ** [μrad ** **]**	120.8	237.5	—	—	—	—
${∆}_{u}$ ** [μm ** **]**	351.22	349.55	—	—	—	—
$f_{1}$ ** [μm ** **]**	2812.02	2365.11	—	—	—	—
$f_{2}$ ** [μm ** **]**	2778.64	2333.44	—	—	—	—
$f_{3}$ ** [μm ** **]**	2729.26	2297.09	—	—	—	—
$u_{0}$ ** [pix ** **]**	3066.2	3186.1	3119.2	3117.7	3098.7	2171.7
$v_{0}$ ** [pix ** **]**	2015.7	2147.1	2084.3	2083.7	2056.3	1608.3
d ** [μm ** **]**	1584.16	1486.54	392.60	522.90	583.80	668.50

**Table 10:** Initial internal parameters for the *HR260-B* dataset and the optimized parameters obtained via the proposed method, Labussière method, Nousias method (for each microlens type: NOUS1, NOUS2, and NOUS3), and Liu method. The oblique line indicates that the calibration failed, and the dashes indicate solutions that did not consider the parameters.

	*HR260-B* (*F* = 105 mm, *H* = 2000 mm)					
	Ours	Labussière	NOUS1	NOUS2	NOUS3	Liu
F **[mm]**	110.766	111.888	58.728	42.712	42.233	57.919
$Q_{1}$ **[$\times 10^{-5}$]**	−7.78	4.38	—	—	—	—
${-Q}_{2}$ **[$\times 10^{-6}$** **]**	−1.224	0.065	—	—	—	—
$Q_{3}$ **[$\times 10^{-8}$]**	−0.730	0.002	—	—	—	—
$P_{1}$ **[$\times 10^{-5}$]**	−9.03	−11.29	—	—	—	—
$-P_{2}$ **[$\times 10^{-5}$]**	2.250	−8.907	—	—	—	—
D ** [mm ** **]**	107.737	106.403	56.692	0.667	58.124	54.857
$-t_{x}$ ** [mm ** **]**	11.13	−7.25	—	—	—	—
$-t_{y}$ ** [mm ** **]**	8.863	16.043	—	—	—	—
${-\theta }_{x}$ ** [μrad ** **]**	689.2	824.7	—	—	—	—
$\theta_{y}$ ** [μrad ** **]**	3087.1	3425.9	—	—	—	—
$\theta_{z}$ ** [μrad ** **]**	276.7	381.2	—	—	—	—
${∆}_{u}$ ** [μm ** **]**	350.27	349.40	—	—	—	—
$f_{1}$ ** [μm ** **]**	2155.54	2588.68	—	—	—	—
$f_{2}$ ** [μm ** **]**	2190.22	2546.87	—	—	—	—
$f_{3}$ ** [μm ** **]**	2225.47	2506.37	—	—	—	—
$u_{0}$ ** [pix ** **]**	3134.5	8168.1	3121.9	3119.5	3122.1	1909.2
$v_{0}$ ** [pix ** **]**	1696.6	−280.7	2086.7	2088.6	2085.2	1687.9
d ** [μm ** **]**	1354.74	1639.79	526.20	1.10	2203.80	686.00

**Table 11:** Runtime comparison (in seconds) between the proposed method and Labussière et al. [20] on four datasets.

Dataset	Method	Corner Extraction	Center Ray Optimization (Iterations, Time)	Extrinsic Optimization (Iterations, Time)	Global Nonlinear Optimization (Iterations, Time)
*R12-A*	Labussière	1027	/	/	(160, 451.26)
	Ours	1041	(6, 8.88)	(98, 20.69)	(152, 378.89)
*R12-B*	Labussière	982	/	/	(100, 284.58)
	Ours	1045	(6, 8.82)	(74, 8.78)	(160, 197.75)
*HR260-A*	Labussière	4824	/	/	(77, 101.68)
	Ours	5069	(5, 0.92)	(250, 19.86)	(93, 64.45)
*HR260-B*	Labussière	4809	/	/	(42, 52.70)
	Ours	4970	(5, 0.91)	(180, 14.45)	(57, 65.48)

## Data Availability

The original contributions presented in this study are included in the article. Further inquiries can be directed to the corresponding author.
